# On the growth impact of different eco-innovation business strategies

**DOI:** 10.1007/s40888-022-00263-x

**Published:** 2022-04-07

**Authors:** Serenella Caravella, Francesco Crespi

**Affiliations:** 1 Roma Tre University, Rome, Italy; 2 BRICK, Collegio Carlo Alberto, Turin, Italy; 3 Department of Economics, Roma Tre University, Rome, Italy

**Keywords:** Firms’ growth, Environmental innovations, Eco-innovation modes, Cluster analysis, Quantile regressions, H32, O44, Q52, J21

## Abstract

The paper investigates whether standard innovation and different types of eco-innovation activities have different effects on firm employment growth. Heterogeneity in terms of growth effects is analyzed by considering how the influence of different innovation strategies varies across firms grouped by their pace of growth. Relying on a sample of 3000 Italian manufacturing firms observed between 2012 and 2016, we find that innovative companies tend to grow faster than their non-innovative counterparts. However, when distinct paces of growth are taken into account, the employment growth impact of innovation will depend on the specific types of innovation strategies pursued by companies. In particular, more complex eco-innovation activities have no impact on employment growth for high-growth firms. This finding suggests that, on average, high-growth firms tend to be too small and too young to be able to expand through more complex EI strategies which, to be handled, require a wide range of cognitive, technological, and financial capabilities.

**Supplementary Information:**

The online version contains supplementary material available at 10.1007/s40888-022-00263-x.

## Introduction^1^

In the current context of economic and employment effects related to the Covid-19 pandemic, understanding the growth potential of Eco-Innovation (EI) activities at the firm level is of the utmost importance. The substantial brake put on many economic and social activities in an attempt to flatten the rise in contagion has left millions of people without work. To protect jobs, incomes, and productive capacity, almost all governments around the world are allocating significant amounts of public resources, part of which are to be used for a global ‘green recovery’.^2^ In particular, the European Union launched the NextGenerationEU plan that shapes the economic recovery of Europe along the *twin transition* to a climate-neutral and digital Europe (EU, 2020). With respect to the ecological goal, 37% of NextGenerationEU budget will be invested in the European Green Deal (EGD) objectives, with about €227 billion to be allocated in renewables, clean hydrogen, and sustainable mobility^3^ in the next 6 years. By leveraging business dynamics directly and indirectly related to the ‘green-economy’, this additional flow of resources is expected to be an effective stimulus for green innovation investment, both on the demand and on the supply sides, with positive implications for the creation of new capital flows, a cleaner environment, a better quality of life and, above all, new jobs (Jacobson et al., 2019).

In such EGD policy framework, private actors are key players in supporting economic recovery along the sustainability path (Markard & Rosenbloom, 2020; Mazzanti, 2018). Through their willingness to take risks, be innovative, and exploit new market opportunities, firms are in a position to extract value from green innovation strategies in order to achieve the goal of expanding their business, hence their productive and employment base.

Yet exploiting the growth-promoting role of EI is not uncomplicated. To be generated, EI calls for the combination of highly diversified and advanced sets of competences (Orsatti et al., 2020; Zeppini & van der Bergh, 2011) which enable the full exploitation of the problem-solving potential of existing and/or new technological trajectories (Quatraro & Scandura, 2019; Barbieri et al., 2020; Santoalha et al., 2021). Therefore, EI represents a major technological effort to be pursued by firms (Carrillo-Hermosilla et al., 2010; Ghisetti et al., 2015) as it is, on average, more likely to be associated with advanced knowledge bases which require the accumulation of knowledge capabilities through experimentation and learning.

Moreover, EI is characterized by a high degree of heterogeneity in firms’ innovation behaviors, which makes its potential as a growth driver even more unpredictable. As recently highlighted by empirical studies, there are distinct ‘modes’ of dealing with EI in terms of engagement (Marin et al., 2015), technological trajectories (Marzucchi & Montresor, 2017), and environmental goals achieved (Castellacci & Lie, 2017; Caravella & Crespi, 2020a). This implies that, among the whole array of EI activities, the most complex and risky ones can hardly be managed by those companies which are particularly weak in terms of knowledge, technological, and financial endowments. This is especially true for small and young firms which, compared to their bigger and experienced peers, generally lack enough cognitive, technical and financial assets to successfully handle multifaced innovation activities (Leoncini et al., 2017; Triguero et al., 2017). Unfortunately, this may result in a major obstacle to the achievement of employment goals, since the greatest incidence of total job creation is related to small and young high-growth companies (Flachenecker et al., 2020).

In comparison to standard innovation, literature on the growth-enhancing role of EIs is still scarcely developed, with very few studies examining their impact by pace of growth, and almost no evidence regarding potential differences in EI behaviours. In order to fill these gaps, the present paper draws attention to the need for a better assessment of the actual potential of different EI strategies to sustain firm growth. An original analytical framework is developed wherein heterogeneity among eco-innovators and employment growth distribution aspects are jointly accounted for. The study relies on data obtained from the 2012–2014 Community Innovation Survey, which is the only source of detailed information on the specific environmental innovation trajectories followed by firms. Focusing on the Italian case, the impact of EI on subsequent 2014–2016 employment dynamics, retrieved by the Asia-Istat database, is estimated for 3000 manufacturing firms according to their innovation trajectories. Empirical evidence suggests that companies at the top quantiles of the growth distribution, being typically small and young and therefore less equipped in terms of resources and capabilities, fail to exploit the growth potential of more complex innovation patterns such as those related to material and energy reduction.

The remainder of the paper is organized as follows. Section 2 reviews previous relevant literature and sketches the research hypothesis. Section 3 presents the data and the empirical strategy before examining the results. Section 4 draws the conclusions by discussing business strategy and policy implications.

## Background literature and research hypotheses

The extensive literature on the role of innovation as a determinant of firm growth mainly concludes that a positive link exists between innovative activities and job creation, though some distinctions are drawn between the effects of product and process innovations (Calvino & Virgillito, 2018; Pianta & Vivarelli, 2003; Vivarelli, 2013, 2014). Specifically, innovation fuels growth via three main channels: first, through cost-saving and productivity gains enabled by new process innovations; second, through additional demand flows stemming from product innovations and complementary goods; third, by providing additional resources investment from the ‘extra-returns’ generated by the introduction of innovations.

The relationship between innovation and job creation at the firm level has been more recently investigated by means of quantile regressions, which show a moderating role of age and size in shaping the conditions under which firms increase their workforce out of their innovative efforts (Coad & Rao, 2008; Segarra & Teruel, 2014; Mazzucato & Parris, 2015; Coad et al., 2016; Bianchini et al., 2018; Colombelli et al., 2019; Siepel et al., 2021). According to this literature, for younger and smaller companies the effect played by innovation activities increases when they are experiencing higher growth performance. This is due to a combination of attributes that allow high-growth firms (HGFs) to better exploit the growth opportunities provided by technological innovation. Given their smaller size, innovation in HGFs is less dependent on internal R&D activities and more frequently associated with cooperative activities and/or based on knowledge spillovers effects (Piergiovanni et al., 1997; Ganotakis & Love, 2011). Furthermore, younger HGFs are more prone to commercialize their innovations and to undertake riskier innovation activities than their bigger peers which, conversely, are more affected by organizational inertia and learning impediments (Criscuolo et al., 2012; Majumdar, 1997).

A relevant implication of these results is that targeted policies to sustain innovation in HGFs are expected to magnify the employment growth potential of innovation. Indeed, HGFs are responsible for the majority of net employment growth. Despite representing around 11% of European firms in the business economy, HGFs were responsible for about 53% of the employment net increase from 2015 to 2016 (Flachenecker et al., 2020). This result is in line with other findings in the literature. For example, relying on a large sample of firm-level employment data for the period 2004–2015 for the 28 EU Member States, Hallak and Harasztosi (2019) show that, with nearly 60%, HGFs exhibit the largest incidence on total job creation. This is basically explained by the young age of these organizations that, in comparison to more mature businesses, are more likely to grow than to downsize and exit. Still analyzing the European context, Ferrando et al. (2019) find that 44% of the total new job creation between 2003 and 2016 was due to HGFs with strong innovative profiles.

When eco-innovation is under scrutiny, the picture becomes less clear. The role of EI innovation as growth lever is scarcely and inconclusively investigated through micro-level studies which detect, according to the case and the proxy used as green innovation measurements (input/output measures from survey data, green patents, environmentally oriented investments, etc.), either positive (Antonietti & Marzucchi, 2014; Gagliardi et al., 2016; Licht & Peters, 2014; Pfeiffer & Rennings, 2001; Rennings et al., 2004), neutral (Rennings & Zwick, 2002), or even negative (Cainelli et al., 2011) effects. Moreover, with very few exceptions (Colombelli et al., 2019; Leoncini et al., 2017), almost no study pays attention to the asymmetric effect of EI on upsized and downsized firms’ ability to grow.

The difficulty experienced with estimating the pro-growth role of EIs mostly depends on the peculiarities of such innovations in terms of both complexity and heterogeneity.

Regarding the former, a growing body of theoretical and empirical literature has highlighted the inner complexity of EI activities with respect to standard ones. At macro-level, green innovations have been found, on average, to be associated to a greater degree of technological novelty as they are more likely to arise from combinations of new and/or existing technologies that belong to ‘distant’ knowledge fields (Barbieri et al., 2020; Messeni Petruzzelli et al., 2011). For these reasons, experience in and capacity for the management of different knowledge capabilities are considered especially important for the successful generation of EI (Orsatti et al., 2020). There exist many analyses supporting this evidence also at the micro-level. First, firms’ capacity to introduce EI activities strongly relies on persistent R&D activities, which represent an even more critical driver than they are for other innovations (De Marchi, 2012; Horbach, 2008; Rennings et al., 2006). On the other hand, since EIs are typically systemic (Andersen, 2002; Foxon & Andersen, 2009) and thus more cooperation-based (De Marchi, 2012), internal investments in green-specific resources have to be complemented with knowledge and competences from external sources (Andersen, 2002; Mancinelli & Mazzanti, 2009; De Marchi, 2012; De Marchi & Grandinetti, 2013; Horbach et al., 2013). Hence, compared to other types of innovations, EI is more likely to emerge from a collective invention dynamic involving high cooperative effort based on mutual relationships with other actors (universities and other research organizations, NGOs, public authorities and other firms in the same or similar sectors). In this context, the capacity of extracting value from EI largely depends on firms’ ability to exploit formal training and learning channels for developing environmental technologies and assimilating those coming from others. Thus, more often than in other technological domains, the kind of resource needed to undertake green innovations may require large-scale conditions as EI-related projects are usually more complex. Moreover, EI is surrounded by increasing technological uncertainty typically associated with innovations that are at the early stages of their technology lifecycle. To be handled, EI requires firms to be equipped with both high managerial abilities and, not less relevantly, a sufficient endowment of financial resources. All these features are typically associated with more mature bigger companies and represent a precondition to fruitfully introducing green-related innovation and pursuing business growth via EI activities. On the contrary, if HGFs are characterized by smaller sizes and younger ages, they are likely to be less experienced in developing the numerous qualities needed for succeeding in EI, i.e. a strong and persistent internal knowledge base, mutual learning networks, scale economies, and easy access to external finance. These issues have been proven to be relevant in the context of the patent-based analysis by Leoncini et al. (2017), one of the few studies assessing the link between EI and firm growth by considering different paces of growth. This study shows that, compared to non-green patents, the employment impact of green patents is higher only for medium-growing firms, suggesting that, because of the inner complexity and costly nature of EI, only relatively established firms are able to benefit from the introduction of green technologies. More recently, by looking at the whole population of SMEs (5100 SMEs across 28 European countries in 2016), Demirel and Danisman (2019) have focused on circular economy eco‐innovations and found that the majority of these fail to boost growth. The reason is that a significant threshold level of investment (i.e. higher than 10% of revenues) in circular eco‐innovations is required for SMEs to derive benefit from it, which highlights how financial constraint represents one of the major factors hampering environmental innovation activities. This is particularly true in the case of HGFs that are, as pointed out by Ferrando et al. (2019), strongly financially constrained.

Building on these arguments, and considering the potentially different impact of standard and eco-innovation on business growth and employment dynamics, we formulate the following research hypotheses:

**HP1**. *Given its major complexity, the pro-growth role of EI is less certain than that of standard innovation, especially for high-growth firms (HGFs)*.

A second issue deserving investigation is heterogeneity in innovation strategies in the domain of environmentally friendly technologies, which may influence their impact on the employment dynamics of firms. Little is known about the differentiated growth potential of environmental innovation. Such limited evidence could be ascribed to a lack of detailed data at the firm level, which has so far confined the empirical efforts of scholars to a generic distinction between *end-of-pipe* vs *clean technologies*. Both types of innovation aim at mitigating the environmental burden of production; however, whereas the former reduces harmful substances that occur as a by-product of production through the adoption of add-on measures (e.g. incineration plants for waste disposal, waste-water treatment plants for water protection, sound absorbers, exhaust-gas cleaning equipment for air-quality control, etc.), the latter alleviates the environmentally harmful impact at the source by substituting or modifying less clean technologies (e.g. recirculation of materials, use of environmentally friendly materials, modification of the combustion chambre design, etc.).

This substantial difference explains why clean technologies tend to overperform *end-of-pipe* technologies not only in terms of environmental benefits but also in terms of economic performances.

More specifically, incremental innovations (i.e. *end-of-pipe* technologies) may display a negative effect on employment due to the fact that their adoption, which is mainly fostered by environmental regulations, may imply relevant costs that, in turn, could lead firms to adopt complementary strategies in order to keep profit margins unchanged. These may consist in labor-saving strategies or output prices increases which would cause employment reductions either directly (the former) or indirectly (the latter), as they potentially lead to output contraction as a consequence of lower demand levels (Costantini et al., 2018; Hazilla & Kopp, 1990).

Conversely, in line with the so-called *win–win* argument, proactive environmental strategies (i.e. those associated with cleaner technologies) can exert a beneficial impact on occupation. In this specific case, cost reductions arising from a reduced use of energy and other materials would result in a complementarity effect between environmental and labor productivity that can positively affect firms’ growth also in terms of jobs created.

The distinction between different eco-innovation patterns has started to be recognized and specifically analyzed by the literature. One of the key contributions is provided by Marzucchi and Montresor (2017), who discover differentiated compositions of Science-Technology Innovation (STI) and Doing Users Innovation (DUI) associated with distinct EI targets. By incorporating new dimensions into the EI phenomenon, this study paves the way for a discussion of heterogeneity among EI innovators, providing empirical support for more sophisticated conceptual schemes (Chesbrough et al., 2006). Through these new lenses, EI innovation is seen as a multiform process made up of a variety of potential patterns and purposes that depend on a plurality of technological trajectories (Castellacci & Lie, 2017; Kiefer et al., 2019; Thurner & Roud, 2016) and combinations of different forms of knowledge (Marzucchi & Montresor, 2017). Following this line of research, further attempts to structure the analysis of different EI patterns are provided by Marin et al (2015) for EU countries, Thurner and Roud (2016) for Russia, Castellacci and Lie (2017) for Korea, Caravella and Crespi (2020a) for Italy. This new stream of literature shows how firms’ EI activities may be highly differentiated in terms of intensity (Marin et al., 2015), originality (Roud, 2018), and green objectives (Caravella & Crespi, 2020a, 2020b; Castellacci & Lie, 2017). Depending on the EI trajectory followed, firms need to be equipped with a different set of capabilities (Yong et al., 2020). However, building the necessary competences may imply different levels of effort depending on the complexity of the environmental strategy adopted. For example, compared to incremental innovations, the implementation of more complex and radical green innovations often requires extra knowledge and skills with respect to the standard knowledge base characterizing a specific industry. Hence, for those EI activities that require a higher level of internal and external capabilities, the growth-enhancing potential of EI strongly depends on a firm’s knowledge and financial resources: two conditions affected by the idiosyncratic characteristics of firms in terms of age and size. In particular, young and small HGFs might not have adequate human capital, technological and financial resources to leverage the payoffs in terms of employment growth linked to more complex EI strategies.

Building on these considerations, we then propose our second hypothesis to be tested:

**HP2**. *The employment impact of different EI strategies might vary according to their differentiated level of complexity, with more complex EI strategies displaying a lower growth-enhancing effect for HGFs.*

## Empirical analysis

### Data

The present analysis is based on a longitudinal dataset obtained by combining data from two different sources: (i) the 2012–2014 Italian Community Innovation Survey (CIS) which, compared to the previous and subsequent CIS waves, allows for an investigation of firms’ environmental innovation strategies through the collection of information on a wide range of aspects related to both standard and environmental innovative trajectories as well as firm-level characteristics^4^; (ii) the ASIA database of the Italian National Statistical Office (ISTAT) that provides information about growth dynamics during the time frame 2014–2016.

Firms are selected from a sample of 5284 manufacturing companies from the 2012 to 2014 CIS database according to the criterion that they should have survived until 2016. Focusing on surviving companies minimizes unobserved heterogeneity, allowing for the identification of a more homogenous population of companies, all displaying some ability to keep existing in the market. Given the nature of the study, survival bias is to be expected, and thus findings on the role of distinct innovation behaviors in the firms’ employment performance must be carefully interpreted. However, this is not considered problematic as, despite the potential for survivor bias, the sample is specifically drawn to examine the drivers of different paces of growth. A quantile regression (QR) framework is adopted, which implies that the estimates along different quantiles of the distribution of the growth rates refer to firms with similar paces of growth. By disentangling the relationships between regressors and firm growth at different quantiles of the growth distribution, QR is preferable to OLS as it estimates the potential heterogeneous impact of covariates on the dependent variable, which would otherwise be observable only by means of conditional mean (Koenker, 2005).

Furthermore, we excluded ‘severe’ outliers based on having the value of the outcome variable (i.e. the logarithmic difference between employment levels in 2014 and 2016) three standard greater than the third quartile or smaller than the first quartile. Hence, the final sample is made up of 3000 manufacturing firms observed during the period 2012–2016.

### The model

To address our research questions, we opt for a growth version of the original logarithmic representation of Gibrat’s Law enriched by a quantile regression (QR) approach (Coad & Rao, 2008; Kesidou & Demirel, 2012).

The model is expressed as

1 $${Growth{(Y)}_{i,t}=lnY }_{i,2016}- {lnY }_{i,2014}= {\alpha }_{0}+{\mathrm{ln}(Y }_{i,2014}) {+\beta }_{\theta }{X}_{i,2012-2014}^{^{\prime}}+{\varepsilon }_{it\theta }$$

The quantile regression coefficient estimates ${\beta }_{\theta }$ solve the following minimization problem for ρ:

2 $$\mathrm{min}\left(\beta \right)\left[\sum_{i=1}^{n}{\rho }_{\theta }(\Delta \mathrm{ln}{\left(Y\right)}_{i,t}-{\beta }_{\theta }{X}_{i,2012-2014 } )\right]$$

where $${\rho }_{\theta }\left(\mu \right)={\theta }_{\mu }$$ if μ = 0, otherwise $${\rho }_{\theta }\left(\mu \right)=(\theta -{1)}_{\mu }$$ if μ < 0.where $$lGrowth{(Y)}_{i,t}$$ is the growth rate of employment calculated as the logarithmic difference between employment in 2016 ( $${Y }_{i,2016})$$ and employement in 2014 $${(Y }_{i,2014})$$ and $${X}_{i,2012-2014}^{^{\prime}}$$ is the set of regressors. As recognized by the literature, among the list of possible growth indicators^5^ (assets, employment, market share, physical output, profits, sales), employment dynamics address some relevant methodological issues (Delmar et al., 2003). First, employment does not require intra-industry comparisons between firms with the same product range, which are needed when market share and physical output indicators are adopted instead. In addition, when compared with other indicators such as asset value and profits, employment is relatively insensitive to both capital intensity and the degree of integration of the industry. Finally, in contrast to sales dynamics, employment is insensitive to inflation and currency exchange rates.

Concerning the regressors, our main variable is categorical and grasps different technological trajectories (one standard and four environmental innovation strategies) carried out by the 7th CIS surveyed firms, with the baseline represented by non-innovators (n = 1410).^6^ In particular, for the identification of the four different EI modes we rely on the taxonomy proposed by Caravella and Crespi (2020a). The authors perform a cluster analysis starting from the CIS information on the different green goals achieved by firms through their innovation-related activities (Table 1).

**Table 1:** Ten types of environmental goals

Variable	
ECOMAT	Reduced material use per unit of output produced
ECOENO	Reduced energy use or ENERGY 'footprint' by firm
ECOPOL	Reduced air, water, noise or soil pollution related to the production
ECOSUB	Replaced materials with less polluting or hazardous substitutes
ECOREP	Replaced fossil energy with renewable energy sources
ECOREC	Recycled waste, water, or materials related to the production
ECOENU	Reduced energy use or ENERGY 'footprint' by the end user
ECOPOS	Reduced air, water, noise or soil pollution by the end user
ECOREA	Recycling of product after use by the end user
ECOEXT	Extended product life through more durable products

**Fig. 1:**
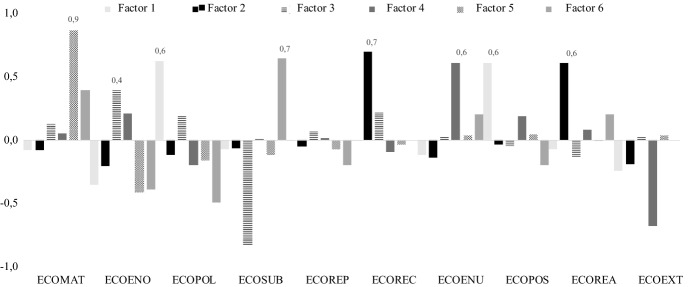
Results of factor analysis (factor loadings). Extraction Method: Principal Component Analysis. Rotation Method: Varimax with Kaiser Normalization. The numbers in bold indicate the variables that are more strongly correlated to each principal component

Due to the high correlation between the ten initial variables,^7^ the clustering analysis is preceded by a Principal Component Analysis (PCA) aimed at obtaining a smaller set of independent (orthogonal) factors. The five extracted factors explain 75.5% of the variance of the whole sample. As shown in Fig. 1, factor 1 assumes very high values for pollution-reducing activities (ECOPOL and ECOPOS); factor 2 mainly refers to recycling innovations (ECOREC and ECOREA); factor 3 combines both process and product energy-saving innovations (ECOENU and ECOENO); factor 5 has a very high loading on the indicator referring to material-reducing innovations (ECOMAT); factor 6 is mostly explained by the variable referring to the replacement of a share of fossil energy with renewable energy sources (ECOREP).

The above six principal components are exploited to cluster firms according to the EI mode they use. The cluster analysis is made up of two steps: a hierarchical cluster to choose the optimal number of groups and a *k-means* cluster based on the clustering method of complete linkage (which minimizes the within-cluster distance between observations) to assign firms to cluster. Using the results of the cluster analysis, environmental innovation strategies are systematized as follows:

(i) *Pollution-reducing EI*. This mode is followed by 276 firms that attach great importance to environmental policies. Relative to other environmental innovators, these companies are found to be smaller and to belong to a specific group.
(ii) *Recycling EI*. This group include 332 firms which have recycling goals at both process and product level and which acknowledge demand-side innovation policies (e.g. green public purchasing) as main policy drivers. These innovators appear bigger in size and do not usually belong to groups.
(iii) *Energy-saving EI*. For this group made up of 126 firms, the crucial policy driver is represented by financial support by governments (supply-side innovation policy). On the contrary, energy-improving innovations are not fueled by green public purchasing and environmental standards impositions. Cost-saving motivations are also of particular importance to this cluster.
(iv) *Material-substituting EI*. This mode is followed by 308 firm that attach little or null importance to policy drivers. Companies in this cluster are less likely to cooperate with external partners and less motivated by voluntary code schemes.

We also account for standard innovation (*SI*), which involves 548 firms introducing technological (process and/or product) innovations not oriented to the achievement of environmental benefits.

A set of control variables that are often included in growth rate regression models is added. Namely, the model controls for: (i) a measurement of average size in terms of number of employees of firm *i* (*lSize 2012–2014*) expressed in logarithm, used to account for the implications of Gibrat’s law (i.e. the trend of size expansion followed by a given firm is independent from its starting size); (ii) the average value added of firm *i* (*lValue added 2012–2014*) in logarithmic terms to consider firm productivity performance based on the argumentation that, only after achieving a certain level of productivity, companies are able to put more emphasis on strategic development and expanding goals (Du & Temouri, 2015); (iii) the growth rate of turnover from 2012 to 2014 (*Market demand 2012–2014*) measured as the logarithmic difference of turnover and used as a proxy for market demand (Horbach & Rennings, 2013) to account for a *demand-pull* growth impulse; (iv) a continuous variable representing firm age transformed in logarithmic terms (*lAge 2012–2014*) to include a wide range of competitive advantages related to experience (Leoncini et al., 2017); (v) an ordinal variable (*Market size 2012–2014*) capturing the local, national, European, and/or extra-EU dimension of the market in which a firm operates and competes; (vi) an ordinal variable referring to the percentage of employees with tertiary degree (*EMPUD 2012–2014)*, considering that higher skilled labor tends to benefit more from growth opportunities experience as it is more likely to achieve higher productivity levels (Syverson, 2011). Finally, we add a group belonging dummy (*GP 2012–2014*) to consider the potential growth-driving effect of group membership, as this may involve an increased value of affiliated firms (Elango et al., 2016) and/or the replication of successful business models and management practices experienced by other affiliated units (Battisti & Lona, 2009). We also include a set of industry^8^ and NUTS2 dummies. Definitions and descriptive statistics of the variables employed in the empirical exercise are reported in Table 2, with Table 3 displaying the bivariate correlations of the variables considered in the analysis. No indication of significant multi-collinearity among the independent variables is found. Furthermore, the variance inflation factor (VIF) ranges from 1.06 to 2.86, well below the threshold level of 5.

**Table 2:** Descriptive statistics (n = 3000)

	Mean	Median	Min	Max	SD
*Dependent variable*					
Empl growth 2014–2016 (log. difference)	0.01	0.0005	− 0.92	0.69	0.154
*Independent variables*					
1.Non innovators 2012–2014 (categorical, *beseline*)	0.47	0	0	1	0.499
2.SI 2012–2014 (categorical)	0.18	0	0	1	0.386
3.Pollution-reducing EI 2012–2014 (categorical)	0.09	0	0	1	0.289
4.Recycling EI 2012–2014 (categorical)	0.11	0	0	1	0.314
5.Energy-saving EI 2012–2014 (dummy)	0.04	0	0	1	0.201
6.Material-substituting EI 2012–2014 (dummy)	0.10	0	0	1	0.304
7.Size 2012–2014 (number of employees)	162	53	10	7.255	361.30
8.Value added 2012–2014 (EUR)	273,847	184,975	12,330	2,75e + 07	698,207
9.Age 2012–2014 (number of years)	33	32	8	133	15
10.Demand growth 2012–2014(log. difference)	0.03	0.03	− 2.39	2.65	0.26
11.Market demand 2012–2014 (ordinal; 1–4)	2.1	2	1	4	0.98
12.GP** 2012–2014 (dummy)	0.53	1	0	1	0.50
13.EMPUD***2012–2014 (ordinal; 1–6)	1.55	1	0	6	1.49

*Expressed in levels; **group belonging; ***percent of employees with tertiary degree

**Table 3:** Correlation matrix (n = 3000)

	1	2	3	4	5	6	7	8	9	10	11	12	13
1	1												
2	− 0.45	1											
3	− 0.30	− 0.15	1										
4	− 0.33	− 0.17	− 0.11	1									
5	− 0.20	− 0.10	− 0.07	− 0.07	1								
6	− 0.32	− 0.16	− 0.11	− 0.12	− 0.07	1							
7	− 0.42	− 0.01	0.21	0.22	0.06	0.23	1						
8	− 0.23	0.01	0.12	0.13	0.06	0.09	0.38	1					
9	− 0.11	0.00	0.09	0.03	0.00	0.05	0.21	0.15	1				
10	− 0.04	0.01	0.01	0.02	0.00	0.01	0.00	0.12	− 0.09	1			
11	− 0.31	0.06	0.12	0.12	0.08	0.14	0.44	0.30	0.09	0.04	1		
12	− 0.30	0.03	0.17	0.13	0.05	0.13	0.60	0.39	0.08	− 0.02	0.34	1	
13	− 0.33	0.06	0.14	0.11	0.10	0.15	0.48	0.36	0.08	− 0.01	0.33	0.40	1

*Variables*: (1) Non-innovators 2012–2014; (2) SI 2012–2014; (3) Pollution-reducing EI 2012–2014; (4) Recycling EI 2012–2014; (5) Energy-saving EI 2012–2014; (6) Material-substituting EI 2012–2014; (7) lSize 2012–2014; (8) lValue added 2012–2014; (9) lAge 2012–2014; (10) Market demand 2012–2014; (11) Market size 2012–2014; (12) GP 2012–2014; (13) EMPUD 2012–2014

Table 4 reports the distribution of observations and innovative trajectories by initial size, sector, and quantile of the growth distribution. Table 5 shows the average values of our dependent variable by initial size, sector, and quantile of the growth distribution for different innovative trajectories during the considered period. Compared to their non-innovative peers, innovative firms pursuing any kind of innovation trajectory during 2012–2014 tend to grow, on average, substantially faster (or to shrink more slowly) in the following three-year period. This evidence is common to all size classes and to most sectors, the only exception being sector CB (textiles, apparel, leather, and related products). Interestingly, when distinguishing across quantiles of the growth distribution, we observe a below- average growth rate of employment for innovative firms in the top-quantiles, whose growth pace is slower than the one recorded by the fastest group of non-innovative companies.

**Table 4:** Distribution of innovative status by size and sectors

Size/sector/quantile	Non innovators	Standard innovators	EI Pollution reducing	EI recycling	EI energy saving	EI material substituting	Total
10–50 empl	66.51	17.26	3.58	5.64	2.96	4.06	1454
51–250 empl	36.33	21.36	11.87	12.18	5.68	12.59	969
251 + empl	15.77	15.60	18.89	22.88	4.85	22.01	577
CA	52.11	18.66	9.15	6.69	3.87	9.51	284
CB	57.53	21.10	6.85	6.58	2.47	5.48	365
CC	62.17	15.54	4.99	11.14	2.93	3.23	341
CD-CG	42.91	14.54	10.28	14.01	4.61	13.65	564
CH	47.88	17.82	10.91	8.91	3.79	10.69	449
CI-CL	28.45	19.08	12.61	15.84	6.47	17.55	587
CM	52.68	22.20	6.59	9.51	3.66	5.37	410
1	56.69	16.86	6.45	6.45	3.80	9.75	605
2	50.73	16.20	8.72	11.96	3.58	8.83	895
3	31.33	14.33	16.33	18.67	6.33	13.00	300
4	45.25	20.66	9.02	10.82	4.26	10.00	610
5	41.19	22.37	9.32	10.85	4.41	11.86	590
Total	410	548	276	332	126	308	3000

CA—food products, beverages, and tobacco products; CB—textiles, apparel, leather, and related products; CC—wood and paper products, and printing; CD-CG— coke, and refined petroleum products; chemicals and chemical products; pharmaceuticals, medicinal chemical, and botanical products; rubber and plastics products, and other non-metallic mineral products; CH—basic metals and fabricated metal products, except machinery and equipment; CI-CL—computer, electronic, and optical products; electrical equipment; machinery and equipment; transport equipment; CM—other manufacturing, and repair and installation of machinery and equipment

**Table 5:** Growth in employment by innovative status and size/sector/quantile

Size/sector/quantile	Non innovators	Standard innovators	EI Pollution reducing	EI recycling	EI energy saving	EI material substituting	Total
10–50 empl	0.004	0.043	0.034	0.065	0.079	0.054	1454
51–250 empl	− 0.001	0.029	0.043	0.021	0.016	0.038	969
251 + empl	− 0.061	− 0.005	− 0.012	0.008	− 0.010	− 0.009	577
CA	0.026	0.049	0.043	0.070	0.025	0.095	284
CB	0.002	0.003	0.014	0.038	− 0.010	− 0.001	365
CC	− 0.014	0.027	0.083	0.017	0.005	0.024	341
CD-CG	− 0.017	0.016	− 0.001	0.042	0.060	0.012	564
CH	− 0.012	0.037	0.028	0.064	0.032	0.021	449
CI-CL	− 0.006	0.037	0.009	− 0.008	0.030	0.006	587
CM	0.020	0.039	0.021	0.024	0.036	0.060	410
1	− 0.207	− 0.182	− 0.186	− 0.193	− 0.164	− 0.161	605
2	− 0.018	− 0.021	− 0.025	− 0.026	− 0.024	− 0.027	895
3	0.025	0.024	0.021	0.021	0.024	0.024	300
4	0.075	0.073	0.072	0.071	0.074	0.068	610
5	0.223	0.210	0.174	0.208	0.238	0.189	590
**Total**	**410**	**548**	**276**	**332**	**126**	**308**	**3000**

CA—food products, beverages, and tobacco products; CB—textiles, apparel, leather, and related products; CC—wood and paper products, and printing; CD-CG— coke, and refined petroleum products; chemicals and chemical products; pharmaceuticals, medicinal chemical, and botanical products; rubber and plastics products, and other non-metallic mineral products; CH—basic metals and fabricated metal products, except machinery and equipment; CI-CL—computer, electronic, and optical products; electrical equipment; machinery and equipment; transport equipment; CM—other manufacturing, and repair and installation of machinery and equipment

**Fig. 2:**
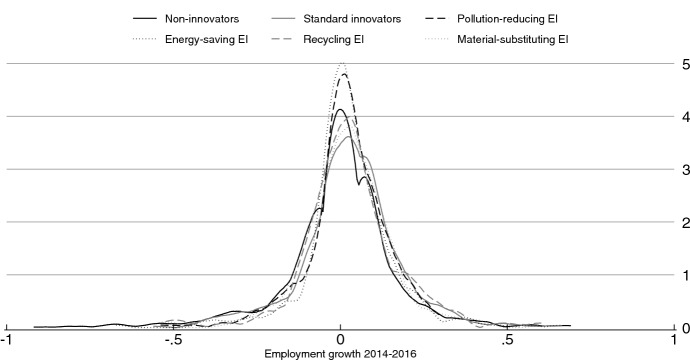
Kernel density of the distribution of employment growth by ‘innovation behaviors’. Growth is calculated in log-differences. Plots obtained using Epanenchnikov kernel

The difference in performance between firms with heterogeneous innovation behaviors is also visible in Fig. 2, where the estimated kernel density of our dependent variable is plotted. Innovative firms’ distribution of employment growth is slightly shifted to the right compared to the one of firms that did not introduce technological innovation in the same period (solid black line). However, when considering the right tail of the growth distribution, innovative firms overlap with non-innovators, implying that the above-average employment growth rate for innovators tends to vanish for faster growth rates. This descriptive evidence suggests that the link between innovation and job creation, as well as the growth premium for innovating firms, are less predictable than expected for companies at the top quantiles of the growth distribution.

### Empirical results

Results are presented in Table 6, where column 1 reports the OLS estimations while columns 2–6 display the output from the quantile regressions. OLS estimates show that both standard innovation and all EI strategies have a positive and significant impact on firms’ growth compared to non-innovating companies. Yet when the two sets of results are compared, OLS estimates tend to hide substantial heterogeneity between different types of innovation activities in terms of their growth-enhancing effects.

**Table 6:** The impact of innovation on employment growth. OLS (model 1) and quantile estimates (models 2–6)

Non innovators 2012–2014	OLS	QR				
	(1)	(2)	(3)	(4)	(5)	(6)
		10th	25th	50th	75th	90th
	Baseline	Baseline	Baseline	Baseline	Baseline	Baseline
SI 2012–2014	0.032*** (0.008)	0.030 (0.018)	0.026*** (0.008)	0.022*** (0.007)	0.029*** (0.008)	0.040*** (0.013)
Pollution-reducing EI 2012–2014	0.031*** (0.009)	0.016 (0.021)	0.024** (0.010)	0.023*** (0.008)	0.027*** (0.010)	0.037** (0.018)
Recycling EI 2012–2014	0.033*** (0.009)	0.051*** (0.016)	0.023*** (0.008)	0.012 (0.008)	0.020** (0.009)	0.033* (0.018)
Energy-saving EI 2012–2014	0.033** (0.013)	0.046** (0.023)	0.007 (0.016)	0.018 (0.011)	0.029 (0.018)	0.048 (0.031)
Material-substituting EI 2012–2014	0.032*** (0.009)	0.040** (0.017)	0.017 (0.011)	0.021*** (0.008)	0.025*** (0.008)	0.011 (0.016)
lSize 2012–2014	− 0.016*** (0.003)	− 0.011* (0.006)	− 0.007** (0.003)	0.012*** (0.002)	0.020*** (0.003)	0.025*** (0.005)
lProductivity 2012–2014	0.032*** (0.005)	0.046*** (0.008)	0.029*** (0.005)	0.021*** (0.004)	0.027*** (0.005)	0.025*** (0.008)
lAge 2012–2014	− 0.013** (0.005)	0.017 (0.011)	0.001 (0.005)	− 0.006 (0.004)	0.023*** (0.006)	0.045*** (0.011)
Market demand 2012–2014	0.139*** (0.016)	0.141*** (0.025)	0.138*** (0.018)	0.144*** (0.015)	0.142*** (0.016)	0.129*** (0.022)
Market size 2012–2014	0.001 (0.003)	0.008 (0.007)	0.007** (0.003)	0.003 (0.003)	− 0.003 (0.003)	− 0.009* (0.005)
GP 2012–2014	− 0.003 (0.007)	− 0.004 (0.014)	− 0.008 (0.008)	− 0.007 (0.006)	0.009 (0.007)	0.015 (0.012)
EMPUD 2012–2014	0.003 (0.002)	0.002 (0.005)	0.004 (0.002)	0.004** (0.002)	0.002 (0.002)	0.006 (0.004)
Regional controls (NUTS-2)	Yes	Yes	Yes	Yes	Yes	Yes
Sectoral controls	Yes	Yes	Yes	Yes	Yes	Yes
N	3000	3000	3000	3000	3000	3000
R2	0.1184					
F	6.84					
Pseudo R2		0.1088	0.0744	0.0577	0.0792	0.1156

Dependent variable: 2014–2016 change in employee headcounts. *p > 0.10. **p > 0.05. ***p > 0.010. Bootstrapped standard errors are reported in parentheses. They are based on 1,000 replications of the data. All regressions include industry affiliation and NUTS-2 of establishment of the national headquarter

In particular, while the growth impact of standard innovation is confirmed for most quantiles (HP1), more differentiated patterns emerge when different eco-innovation modes are considered (HP2). Hence, as argued in Sect. 2, the relationship between growth and different innovation strategies could be mediated by the pace of growth of firms. Concerning controls, our results are in line with the key findings of the recent empirical literature on firm growth (Coad, 2010; Coad et al., 2013). Younger and smaller companies tend to grow faster than their bigger and older counterparts which, in turn, display a more pro-cyclical dynamic over time in terms of turnover and job creation (Ferrando et al., 2019). Thanks to a greater flexibility in adjusting their production and organizational set-ups (Ericson & Pakes, 1995; Jovanovic, 1982), smaller and younger companies better adapt to changes in market conditions. More specifically, the quantile regressions show that smaller companies grow faster across all quantiles, while the growth premium for younger firms is confirmed only for the top two quantiles. This result highlights the relevance of small and young companies within the group of HGFs.

Furthermore, while the effectiveness of both productivity (*lProductivity 2012–2014*) and demand-triggering *(Demand size 2012–2014)* channels in shaping firm employment dynamics is confirmed, the coefficients related to market size *(Market size 2012–2014)* are not statistically significant for most quantiles. An exception is represented by the 90th quantile, where the negative and slightly statistically significant relationship between the dimension of the market and employment growth can be explained by the fact that firms with the fastest paces of growth are too young and small and thus not yet established on international markets. Moreover, the variable representing skilled labor (*EMPUD 2012–2014*) is positive and statistically significant only for the 25th quantile, while the group membership dummy (*GP 2012–2014*) is not statistically significant in any quantiles. Both results suggest that the considered variables are not fully able to grasp their direct growth-enhancing effects when innovation activities and other relevant features at firm level have been already accounted for.

The detailed analysis of the different EI trajectories across quantiles highlights the growth advantage of firms following the *Pollution-reducing EI* mode, whose related coefficient, differently from other EI modes, remains highly statistically significant (p < 0.05) for most quantiles. Being largely achieved through incremental innovation activities associated with lower technical risk (Aghion et al., 2009; Cuerva et al., 2014) and little investment efforts (De Marchi & Grandinetti, 2013), this mode turns out to be the simplest one if environmental regulations are to be fulfilled (Caravella & Crespi, 2020a; Horbach, 2008; Veugelers, 2012). For these reasons, it may be easily introduced by slower firms as well as by fast-growing companies. Consistently with our HP2, the growth advantage attached to other EI strategies is less diffused across quintiles as the complexity associated with different EI activities increases. More in detail, we find that the *Recycling EI* mode is positive (p < 0.05) only for the 10th, 25th and 75th quantiles, while the *Material-substituting EI* coefficient is positively correlated (p < 0.05) with the growth of firms at 10th, 50th and 75th quantiles. Finally, the *Energy-saving EI* variable affects growth positively only in the case of companies belonging to the 10th quantile (p < 0.05). In brief, the majority of EI-related growth premiums vanish or loose statistical significance for the group of HGFs.

The absence of a growth effect displayed by more complex EI modes for faster firms can be related to their idiosyncratic characteristics. On average, the companies included in top quantiles are too small and too young to be sufficiently resource-based to effectively deal with EI’s major complexity (Consoli et al., 2016) and marketability (Leoncini et al., 2017). This happens for the *Energy- saving EI* as well as the *Material-substituting EI.*

In this respect our findings may suggest that the production cost benefits associated with the efficiency gains achieved through these modes are larger and more relevant for shrinking, less-growing, established firms, than for high-growth firms. As shown by Costantini et al. (2018), the introduction of this kind of green innovation is often the result of restructuring processes aimed at reducing production costs rather than achieving production expansion, as suggested by the fact that the positive link between *energy-saving* mode and growth is exclusively detected for firms in the 10th quantile. Moreover, since they have the double aim of reducing pollution and improving efficiency performances, both *energy-saving* and *material-substituting* patterns are expected to involve more complex and relevant investments, and thus require a strong effort in terms of financial and technological capabilities. Indeed, these innovation activities are typically associated with a high incidence of specialized and highly qualified employees, who are able to reorganize production processes in a more resource-efficient way (Horbach & Renning, 2008). This requires advanced knowledge management strategies to recruit and manage internal cognitive resources (Yong et al., 2020), a task that might be particularly challenging for HGFs.

Finally, concerning the *Recycling EI* mode, we find that the increase in product value and the reduction in production costs entailed in this innovation trajectory have a weaker explanatory power (p < 0.10) for the employment growth of HGFs compared to firms in other quantiles (10th, 25th, 75th). As shown by Caravella and Crespi (2020a), this mode, which is framed into the Long-Life-Assessing (LCA) logic, mainly concerns big companies that intercept public green procurement (GPP) flows by introducing LCA-related innovations (Cainelli et al., 2020; Cheng et al., 2018). The reduction in demand uncertainty associated with environmentally-friendly products and services turns out to be less relevant to explaining growth rate differentials of HGFs, which may lack the necessary experience to maximize public procurement related growth opportunities (Caravella & Crespi, 2020b; Divella & Sterlacchini, 2020). Furthermore, as extensively demonstrated by Demirel and Danisman (2019), LCA-related innovations require a 10% threshold investment (in revenues) to deliver employment growth: an amount of funding that financially-constrained firms, as HGFs characteristically are (Ferrando et al., 2019), may have difficulties to manage.

The quantile estimation results hence confirm the presence of heterogeneity in the growth effects of different EI strategies depending on the complexity/heterogeneity of different innovative modes as well as the differentiated patterns of firms’ growth. Indeed, this is not only a signal that firm-specific idiosyncratic characteristics might be correlated with companies’ ability to seize distinct environmental innovation-related growth opportunities. These heterogeneities might in fact also be linked to innovation-specific features such as cognitive and technological capabilities that are relevant for the development of innovation processes, supply of high-level skills, linkages with external networks, access to financial resources.

### Robustness checks

The reliability of our results for HGFs (90th quantile of the employment growth distribution) is further tested through a number of robustness checks based on different specifications of the model (Table 7). With respect to the growth premium associated with *SI 2012–2014,* results tend to be robust to the omissions of control variables (columns 1 and 2), while the estimated return of EI in terms of job creation vanishes for all the considered patterns. On the contrary, no substantial difference in the effect of our variables of interest is found when more detailed dummy variables are added (two-digit NACE rev. 2), when a nonlinear relationship between initial size and employment growth (column 4) is assumed, or when the initial size is expressed in terms of turnover (column 5). Finally, we compare the return of innovation trajectories in terms of job creation with a narrowed *baseline* group of companies which have not introduced any technological innovation but only organizational and/or marketing innovation during 2012–2014 (column 6). The link between innovation and job creation is found to be non-statistically significant in comparison to HGFs characterized by a non-technologically innovative profile. This would suggest that, more in general and differently from organizational and marketing novelties, technological patterns both take longer and require more efforts in terms of experience, knowledge, and financial strength in order to be managed and turned into growth by HGFs.

**Table 7:** Robustness checks: alternative specifications

Non innovators 2012–2014	(1)	(2)	(3)	(4)	(5)	(6)
	No controls	Only dummies	More detailed dummies	Square size	Size: turnover	Comparison with non technological innovators only
	Baseline	Baseline	Baseline	Baseline	Baseline	
SI 2012–2014	0.028* (0.017)	0.033** (0.015)	0.037*** (0.013)	0.037** (0.015)	0.040*** (0.012)	0.022
						(0.034)
Pollution-reducing EI 2012–2014	− 0.009 (0.018)	0.002 (0.015)	0.039** (0.016)	0.025* (0.014)	0.037** (0.017)	0.004
						(0.035)
Recycling EI 2012–2014	0.001 (0.024)	0.022 (0.020)	0.026* (0.013)	0.032 (0.020)	0.033** (0.017)	0.007
						(0.034)
Energy-saving EI 2012–2014	0.044 (0.024)	0.033 (0.040)	0.066 (0.032)	0.046 (0.041)	0.048 (0.032)	0.045
						(0.042)
Material substituting EI 2012–2014	− 0.006 (0.017)	− 0.003 (0.013)	0.019 (0.011)	0.011 (0.013)	0.011 (0.017)	− 0.006
						(0.035)
lSize 2012–2014			− 0.028*** (0.006)			0.025***
						(0.006)
lSize 2012–2014 (squared)				0.002*** (0.001)		
lTurnover 2012–2014					0.025*** (0.005)	
Not technological innovators 2012–2014						*baseline*
lValue added 2012–2014			0.027*** (0.008)	0.024*** (0.007)	0.050*** (0.011)	0.008 (0.010)
lAge 2012–2014			− 0.039*** (0.011)	0.044*** (0.010)	0.045*** (0.012)	0.049*** (0.015)
Market demand 2012–2014			0.132*** (0.025)	0.123*** (0.020)	0.129*** (0.022)	0.159*** (0.031)
Market size 2012–2014			− 0.006 (0.005)	− 0.008 (0.006)	− 0.009** (0.004)	− 0.004 (0.008)
GP 2012–2014			0.017 (0.013)	0.012 (0.013)	0.015 (0.012)	0.033* (0.017)
EMPUD 2012–2014			0.005	0.005 (0.004)	0.006 (0.004)	0.005
						0.022
Regional dummies (NUTS-2)	No	Yes	Yes	Yes	Yes	Yes
Sectoral dummies^*^	No	Yes	No	Yes	Yes	Yes
Sectoral dummies (two-digit)	No	No	Yes	No	No	No
N	3000	3000	3000	3000	3000	1450
Pseudo R2	0.0037	0.0515	0.1252	0.1135	0.1106	0.1531

Dependent variable: 2014–2016 change in employee headcounts, 90th quantile of the distribution. *p > 0.10. **p > 0.05. ***p > 0.010. Bootstrapped standard errors are reported in parentheses. They are based on 1000 replications of the data

## Conclusions

Combining different literature insights, including the empirical ones on firm growth (Coad et al., 2013) as well as the innovation studies on EI complexity (Carrillo-Hermosilla et al., 2010; Ghisetti et al., 2015) and EI heterogeneity (Marzucchi & Montresor, 2017; Castellacci & Lie, 2017; Caravella & Crespi, 2020a), we explored potential heterogeneities in the growth-innovation relationship of different innovation strategies. This was done with respect to the Italian case, whose structural peculiarities make stimulating business growth and understanding the phenomenon particularly urgent. Covering the period 2012–2016, which overlaps with the period following the economic stagnation of 2008, our results must be interpreted in the light of the pre-dated historical pathological weaknesses that affect the Italian productive system. These are due to a number of causes, including a lack of competition in some key strategic sectors, the limited size of financial markets, the ineffectiveness of industrial and innovation policy measures, allocative inefficiency, the historical prevalence of SMEs, the innovation and technology gap, historical disparities between regions, etc. The shortcomings of the Italian economy would explain, on the one hand, the constant growth rate shown by most of the sampled firms (Fig. 1) while motivate, at the same time, the strong interest in providing scientific evidence on the growth-innovation nexus for firms capable of expanding their size. This group, usually referred to as high-growth firms (HGFs), appears particularly restricted in the Italian case. In fact, compared to other EU members, the incidence of HGFs in Italy is particularly low. According to the ‘HGFs indicator framework’ developed by the European Commission in order to capture the most important factors that determine the overall quality of the ecosystem of HGFs, Italy is well below the European average, displaying the lowest score along with Belgium, Cyprus, Greece, and Romania: in the words of the EC, ‘Italian overall performance in factors determining the development of high growth enterprises appears unsatisfactory’ (Flachenecker et al., 2020).

Keeping these considerations in mind, we recognize that our analytical framework is strongly influenced by the peculiarities of the Italian case, and thus results on the role of distinct innovation behaviors in the employment performance of firms could vary for companies based in different country contexts.

Going back to the output of the study, our results confirm the growth potential of standard innovation, while a different picture emerges when different eco-innovation strategies and different firm growth patterns are scrutinized.

Once differentiated paces of growth are considered, the employment-growth potential of eco-innovation for Italian firms emerges to be conditioned to the specific innovation patterns followed. While less complex eco-innovation strategies such as the ones stemming from pollution-reducing objectives are more likely to push companies on a superior growth trajectory with a rather homogeneous effect across quantiles, the growth premium associated with more complex cost-saving and efficiency related motivations (*Energy_saving EI* and *Material_substituting EI*) is only detected for firms displaying slower paces of growth. Introducing these kinds of green technologies help reduce the production costs of companies belonging to the less dynamic (in employment growth terms) groups, but does not fuel the growth of HGFs in general. Due to the high incidence of small and young companies in the group of top-quantiles firms, one may suppose that, on average, HGFs do not possess an adequate level of experience and capabilities to extract value, and thus grow, through more complex innovations channels.

Similar conclusions might explain the low explanatory power of the recycling mode, which is strongly related to public demand. Actually, we find that if the innovation strategy is mainly based on the reuse of materials, the relative growth premium decreases in its statistical significance for HGFs that, being on average smaller and younger, are less capable of intercepting the environmentally-oriented part of public procurement.

Such results carry some relevant managerial and policy implications. Concerning the former, our evidence suggests that the potential complementarity between firms’ innovative and advanced knowledge resources may be crucial to capturing the growth potential of more complex environmental innovation patterns for HGFs. This specific issue appears to deserve more attention from entrepreneurship studies, as the availability of specific competences is highly relevant to leverage the growth effects of eco-innovation activities. In this respect the absorptive capacity of external cognitive resources is a precondition to benefiting from knowledge exchanges between industry and external entities. To this aim, firms should dedicate much effort to the building of competences and networks for taking advantage of knowledge developed by external knowledge-intensive partners, such as KIBS, universities, and other research institutions.

On the policy side, the provided evidence suggests that environmentally-oriented policy innovations can sustain growth processes of both downsized and less growing firms, but they fail to support the further expansion of HGFs, which, indeed, are the major source of total job creation. These findings are particularly relevant to the current European context, where the Green Deal has been launched and environmental innovation is seen as a major instrument to overcome the current unemployment crisis caused by the Covid-19 pandemic.

Future research along these lines could contribute to further study the identified relationships. In particular, longer time series would allow for more sophisticated and robust econometric analysis, while the availability of comparable firm-level data for other European economies would allow for an exploration of relevant country-level heterogeneities.

## Supplementary Information

Below is the link to the electronic supplementary material.

Supplementary file1 (DOCX 20 KB)

## Footnotes

1 This paper has been developed under the research agreement between CENTRO STUDI DELLE CAMERE DI COMMERCIO—GUGLIELMO TAGLIACARNE and the Department of Economics of Roma Tre University. This work was supported by the Italian Ministry of University and Research, Scientific Research Program of National Relevance (PRIN) 2017, project “Innovation for global challenges in a connected world: the role of local resources and socio-economic conditions”.

2 https://www.vivideconomics.com/wp-content/uploads/2021/02/Greennes-of-Stimulus-Index-5th-Edition-FINAL-VERSION-09.02.21.pdf.

3 https://ec.europa.eu/commission/presscorner/detail/en/ip_20_1657.

4 The 2012–2014 Italian CI survey exploited for this analysis provides data on 17,532 firms involved in manufacturing and service sectors. Firms with a minimum of 10 employees are identified through stratified random sampling based on size, sector and geographical coordinates, while a census survey includes all firms employing more than 249 workers. The web-based questionnaire is about 12 pages long and the response rate for wave 7th is 62.8%. In comparison with previous CIS waves, CIS7 represents a step forward in the investigation of firms’ environmental innovation strategies, as it collects information on a wide range of aspects related to EI. More precisely, firms have to report about the type and goals achieved through their environmental innovation. The 2012 CIS questionnaire is available at the link. https://circabc.europa.eu/ui/group/47133480-29c1-4c23-9199-72a631f4fd96/library/32ab7d19-446e-404c-9ea5e2524065b2a0/details.

5 Other indicators of growth have been used by scholars to investigate the economic impact of EI. By looking at the link between regulation, innovation, and firm turnover, Ambec et al. (2013) find that a one-unit increase in environmental regulation stringency leads to an increase of 0.49 in environmental R&D which, in turn, allows turnover to increase by 0.37. However, the direct effect of a one-unit increase of environmental stringency on turnover is found to be negative (− 0.78). Doran and Ryan (2016) adopt the ratio between turnover and employees. Their results show that both generic innovation and generic EI exert a significant, but opposite, impact on growth. In particular, the impact of generic innovation on turnover is found negative, while generic EI appears to positively affect firms' performances. Marin (2014) directs attention to the ratio between value added and employees by providing evidence that green patenting activities lead to a substantially lower return when compared to not-green patents. Finally, Ghisetti and Rennings (2014) investigate the effects of efficiency-improving (EREI) and externality-reducing (EI) innovations on firms ROA and find that EREI innovations are positively and significantly associated with the ROA, while EI innovations exert a negative but weak impact.

6 Some considerable endogeneity concerns can be raised in our estimation framework. First, as a result of an anticipation effect of future technology, firms may decide to enlarge their employment base by, for example, hiring dedicated R&D human resources to be engaged in the development of innovation-enhancing innovations. Second, the possibility that firms may decide to engage in different innovation strategies due to changes in the composition and nature of available labor force cannot be excluded a priori. We try to deal with this potential simultaneity bias turning to 2012–2014 for independent variables (e.g. concluded innovation strategies) and to 2014–2016 for outcome variable, while controlling for initial employment base during 2012–2014.

7 See Appendix for detailed information.

8 The seven sector dummies included are: CA (food products, beverages, and tobacco products); CB (textiles, apparel, leather, and related products); CC (wood and paper products, and printing); CD-CG (coke, and refined petroleum products); chemicals and chemical products; pharmaceuticals, medicinal chemical, and botanical products; rubber and plastics products, and other non-metallic mineral products; CH (basic metals and fabricated metal products, except machinery and equipment); CI-CL (computer, electronic, and optical products); electrical equipment; machinery and equipment; transport equipment; CM (other manufacturing, and repair and installation of machinery and equipment). *(Statistical classification of economic activities in the European Community (NACE) Second Revision*).
