# Supplementary material for: On the growth impact of different eco-innovation business strategies
Source: Econ Polit (Bologna). 2022 Apr 7;39(2):657–83. doi: 10.1007/s40888-022-00263-x (PMC8986970; doi:10.1007/s40888-022-00263-x)
Supplement: Supplementary file 1 — Supplementary file1 (DOCX 20 KB) [file 40888_2022_263_MOESM1_ESM.docx]

**Appendix**

***EI modes identification strategy***

The identification of the distinct EI modes follows the approach proposed by Castellacci and Lie (2017), which consists of a clustering procedure preceded by a Principal Component Analysis. The PCA is carried out on ten variables (Table 1A): six referring to the achievement of environmental benefits experienced within the enterprises through innovation (namely ECOMAT. ECOENO. ECOPOL. ECOSUB. ECOREP. ECOREC) and four referring to the achievement of environmental benefits experienced during the consumption or use of a good or service by the end user through innovation (i.e. ECOENU. ECOPOS. ECOREA. ECOEXT). The sample is composed of 1,807 manufacturing companies reporting to have introduced at least a process or product environmental innovation over the period 2012-2014.

**Table 1A.** Ten types of environmental goals: Descriptive statistics

| Variable |  | | Mean | S. Dev | Min | Max | Freq |
| --- | --- | --- | --- | --- | --- | --- | --- |
| **Environmental benefits obtained within the enterprise** | | |  |  |  |  |  |
| **ECOMAT** | | Reduced material use per unit of output produced | .5307139 | .4991939 | 0 | 1 | 959 |
| **ECOENO** | | Reduced energy use or ENERGY 'footprint' by firm | .71057 | .4536234 | 0 | 1 | 1.002 |
| **ECOPOL** | | Reduced air, water, noise, or soil pollution related to the production | .6043165 | .4891324 | 0 | 1 | 1.350 |
| **ECOSUB** | | Replaced materials with less polluting or hazardous substitutes | .5002767 | .5001383 | 0 | 1 | 1.151 |
| **ECOREP** | | Replaced fossil energy with renewable energy sources | .2047593 | .4036373 | 0 | 1 | 955 |
| **ECOREC** | | Recycled waste, water, or materials related toproduction | .3768677 | .4847355 | 0 | 1 | 982 |
| **Environmental benefits obtained by the end user** | | |  |  |  |  |  |
| **ECOENU** | | Reduced energy use or ENERGY 'footprint' by the end user | .5168788 | .4998534 | 0 | 1 | 827 |
| **ECOPOS** | | Reduced air, water, noise, or soil pollution by the end user | .4360819 | .4960349 | 0 | 1 | 572 |
| **ECOREA** | | Recycling of product after use by the end user | .2999447 | .4583603 | 0 | 1 | 711 |
| **ECOEXT** | | Extended product life through more durable products | .3680133 | .4823985 | 0 | 1 | 665 |

By looking at Table 2A, which reports the total number of environmental goals achieved by EI innovators, we can note a clear trend of complementarity between distinct goals. As also shown by the high pairwise correlations among the ten variables reported in Table 3 (see the next section), the complementarity could be due to the high degree of complexity and closely which is usually associated with green technologies.

**Table 1A.** Number of environmental goals reached by CIS7 manufacturing firm

| # of environmental goals | Freq. | Perc. (%) | Cum. Perc. (%) |
| --- | --- | --- | --- |
| 1 | 152 | 8.41 | 8.41 |
| 2 | 287 | 15.88 | 24.29 |
| 3 | 291 | 16.10 | 40.40 |
| 4 | 252 | 13.95 | 54.34 |
| 5 | 207 | 11.46 | 65.80 |
| 6 | 195 | 10.79 | 76.59 |
| 7 | 172 | 9.52 | 86.11 |
| 8 | 111 | 6.14 | 92.25 |
| 9 | 85 | 4.70 | 96.96 |
| 10 | 55 | 3.04 | 100 |
| Total | 1.807 | 100 |  |

Note: Sample of green innovators only: n =1807

Before performing the Principal Component Analysis, a standardization process is needed to establish if and to what extent a given EI strategy is focused on a specific environmental goal. In more detail, the standardization rule is distinctly applied to two groups of variables: IEB (internal environmental benefits) and EEB (external environmental benefits). For both, each of the six (four) variables referring to the benefits experienced within firms (by the end user) is divided by the total number of environmental goals reached within firms (by the end user) by means of the innovation introduced. Formally:

$${IEB}_{y}=\frac{{IEB}_{y}}{\sum_{i} I{EB}_{y}}$$

$${EEB}_{x}=\frac{{EEB}_{x}}{\sum_{i} E{EB}_{x}}$$

(1)

This standardization rule allows for the identification of distinct EI technological trajectories by assigning higher values to focused innovators with narrow EBs and lower values to those developing broader EI strategies as associated with the achievement of more than one EB. In so doing, environmental innovators are grouped on the basis of the predominance assigned to specific internal and external environmental targets by the innovation strategy carried out.

**Table 4A.** Results from PCA

| Component | Eigenvalue | Difference | Proportion | Cumulative |
| --- | --- | --- | --- | --- |
|  |  |  |  |  |
| **Comp1** | **1.67052** | **.188764** | **0.1671** | **0.1671** |
| **Comp2** | **1.48176** | **.267153** | **0.1482** | **0.3152** |
| **Comp3** | **1.2146** | **.0590894** | **0.1215** | **0.4367** |
| **Comp4** | **1.15552** | **.130689** | **0.1156** | **0.5522** |
| **Comp5** | **1.02483** | **.0216984** | **0.1025** | **0.6547** |
| **Comp6** | **1.00313** | **.103102** | **0.1003** | **0.7550** |
| Comp7 | .900026 | .225697 | 0.0900 | 0.8450 |
| Comp8 | .67433 | .0196632 | 0.0674 | 0.9125 |
| Comp9 | .654666 | .434042 | 0.0655 | 0.9779 |

Note: Factors with eigenvalue higher than 1 extracted. The final factors together explain 75.5% of total variance.

The six components are then exploited in the cluster analysis.
